# Cerebral blood flow and functional connectivity immediate changes following intradermal acupuncture in major depressive disorder

**DOI:** 10.3389/fnins.2026.1805907

**Published:** 2026-04-17

**Authors:** Zelin Yu, Xiaoting Wu, Jiajia Yang, Shaungyi Pei, Jia Wang, Zhijian Cao, Jianqiao Fang, Xiaomei Shao

**Affiliations:** 1 Key Laboratory for Research of Acupuncture Treatment and Transformation of Emotional Diseases, The Third Clinical Medical College, Zhejiang Chinese Medical University, Hangzhou, China; 2 The Third Affiliated Hospital of Zhejiang Chinese Medical University, Hangzhou, China; 3 The Second Affiliated Hospital of Zhejiang University School of Medicine, Hangzhou, China; 4 Department of Radiology, The First Affiliated Hospital of Zhejiang Chinese Medical University, Hangzhou, China; 5 Department of Mental Health, The First Affiliated Hospital of Zhejiang Chinese Medical University, Hangzhou, China

**Keywords:** acupuncture, cerebral blood flow, depression, fMRI, functional connectivity

## Abstract

**Background:**

Acupuncture has been increasingly applied as an adjunctive treatment for major depressive disorder (MDD), yet its central neurobiological mechanisms remain insufficiently understood. Cerebral blood flow (CBF) and functional connectivity strength (FCS) provide complementary perspectives on regional metabolic activity and large-scale functional integration, and their coupling may reflect neurovascular coordination relevant to depression.

**Methods:**

Twenty patients with MDD and twenty matched healthy controls (HC) underwent resting-state MRI. Patients received intradermal acupuncture (IA) and were scanned before and immediately after stimulation, while healthy controls were scanned once. Voxel-wise analyses of CBF, FCS, and their ratio (CBF/FCS) were performed to characterize acupuncture-related changes in neurovascular coupling. Group comparisons and pre–post acupuncture effects were assessed at the whole gray matter level.

**Results:**

Acupuncture induced significant alterations in CBF/FCS coupling across widespread brain regions, including the bilateral precuneus, postcentral gyrus, superior temporal pole, superior frontal gyrus, occipital cortex, and cerebellum. These regions are primarily involved in sensorimotor processing, cognitive control, and emotional regulation. Overall, IA was associated with an immediate increase in CBF/FCS coupling, suggesting enhanced coordination between cerebral perfusion and functional network integration.

**Conclusion:**

This study provides evidence that intradermal acupuncture modulates neurovascular coupling in patients with MDD, offering neuroimaging-based insights into its antidepressant mechanisms. The findings support the notion that acupuncture may facilitate more efficient brain function by optimizing the balance between neural activity and metabolic supply.

## 1 Introduction

Major depressive disorder (MDD), marked by persistent sadness, cognitive dysfunction, and impaired psychosocial functioning, imposes a considerable burden on individuals and healthcare systems worldwide (McCarron et al., 2021; García-Montero et al., 2022). Selective serotonin reuptake inhibitors (SSRIs), which exert antidepressant effects primarily by increasing central 5-hydroxytryptamine (5-HT) availability and promoting neuroplasticity, are recommended as first-line treatments across age groups (Matsushima et al., 2019; Sanchez et al., 2014; Boaden et al., 2020). However, clinical response to initial antidepressant therapy remains suboptimal, with more than two-thirds of patients failing to achieve adequate symptom remission (Jha et al., 2018). Limitations such as delayed therapeutic onset, treatment resistance, relapse, withdrawal symptoms, and adverse effects further highlight the need for complementary or alternative interventions for MDD (Horowitz and Taylor, 2019; Schuch et al., 2016; Carvalho et al., 2016).

Acupuncture has gained increasing attention as a non-pharmacological adjunctive therapy for depressive disorders (Zhao et al., 2019; Yang et al., 2022; Armour et al., 2019). Clinical evidence suggests that acupuncture alleviates depressive symptoms, reduces somatic complaints, and improves sleep quality in patients with MDD. Compared with manual acupuncture, intradermal acupuncture (IA) offers practical advantages, including sustained low-intensity stimulation, ease of administration, and minimal interference with daily activities (Noda et al., 2015; Wang et al., 2021; Chen et al., 2021). Notably, previous work from our group has provided clinical evidence supporting the antidepressant efficacy of intradermal acupuncture (Wu et al., 2025). However, the central neurobiological mechanisms underlying these therapeutic effects remain to be further elucidated.

From a mechanistic perspective, existing pathophysiological models of MDD provide a useful framework for interrogating the central effects of intradermal acupuncture. Among these, the monoaminergic hypothesis—particularly dysfunction of the serotonergic system—has received substantial empirical support (Monroe and Harkness, 2022). The dorsal raphe nucleus (DRN) and median raphe nucleus (MRN), as primary sources of central serotonergic projections, play crucial roles in emotional regulation and antidepressant response. Altered FC patterns involving these nuclei and subcortical regions have been consistently implicated in MDD (Han et al., 2019). Experimental studies further suggest that acupuncture may exert anxiolytic and antidepressant effects by modulating serotonergic signaling within the DRN and MRN, including regulation of serotonin transporter activity and brain-derived neurotrophic factor expression (Yang et al., 2017; Wu et al., 2017). However, direct neuroimaging evidence linking IA to functional alterations in MDD remains limited.

Resting-state cerebral blood flow (CBF) and functional connectivity strength (FCS) provide complementary perspectives on regional metabolic activity and large-scale functional integration. CBF reflects regional cerebral perfusion and metabolic supply, whereas FCS reflects the extent of functional connectivity of a given voxel with the rest of the brain. The relationship between these two measures may provide additional insight into neurovascular coupling, which reflects the coordination between local metabolic support and neural communication efficiency. Increasing evidence suggests that abnormalities in neurovascular coupling may contribute to the pathophysiology of psychiatric disorders, including MDD. Therefore, examining both CBF and FCS may provide a more integrated understanding of the central effects of acupuncture than either measure alone.

Therefore, the present study aimed to investigate the immediate central effects of IA in patients with MDD using resting-state MRI. By combining CBF and FCS analyses, we sought to characterize IA-related changes in cerebral perfusion, functional integration, and their coupling relationship, thereby providing neuroimaging evidence for the central mechanisms of IA in MDD.

## 2 Method

### 2.1 Study design

A total of 40 right-handed participants were enrolled in this study, including 20 patients diagnosed with MDD and 20 age- and sex-matched healthy controls. All participants provided written informed consent prior to participation. The present fMRI study was conducted as a single-center neuroimaging substudy embedded within a multicenter randomized controlled trial. All participants included in the MRI analysis were recruited from The Third Affiliated Hospital of Zhejiang Chinese Medical University (Approval No. ZSLL-KY-2022-001-01-01). The parent trial was conducted from November 2022 to January 2024 and was registered at ClinicalTrials.gov (Identifier: NCT05720637). This neuroimaging substudy was covered by the ethical approvals and trial registration of the parent study. The study was conducted in accordance with the Declaration of Helsinki.

### 2.2 Sample size estimation

The present study was designed as an exploratory single-center neuroimaging substudy embedded within a parent multicenter randomized controlled trial. Therefore, the sample size was determined primarily based on feasibility, participant availability at the imaging center, and reference to previous acupuncture-related fMRI studies with similar exploratory designs. Previous reviews of neuroimaging studies on acupuncture have shown that sample sizes in this field are generally modest, and approximately 20 participants per group is commonly used in exploratory fMRI research and considered acceptable for detecting significant imaging alterations (Wang Z.-N. et al., 2025; Zhang et al., 2021). Accordingly, 20 patients with MDD and 20 healthy controls were included in the present study.

### 2.3 Inclusion and exclusion criteria

The diagnosis of MDD was established by two senior psychiatrist using the Structured Clinical Interview for DSM-5 (Zulauf Logoz, 2014). Symptom severity was assessed with the 17-item Hamilton Depression Rating Scale (HAMD-17) (Hamilton, 1960), and only patients with a total score greater than 17 were included in the MDD group. Healthy control participants were screened using the non-patient edition of the SCID to confirm the absence of any current or past psychiatric disorders.

Exclusion criteria for all participants included contraindications to MRI; major psychotic disorders (eg, schizophrenia, bipolar disorder, or other psychotic disorders); substance-related disorders; severe or unstable medical illnesses; positive suicidal tendency; pregnancy or lactation; inability to cooperate with study procedures; allergy to adhesive tape or fear of intradermal acupuncture; prior intradermal acupuncture treatment; and participation in other clinical trials. For healthy controls, any personal history of psychiatric disorders or a first-degree family history of psychotic disorders was also considered exclusionary. More details are shown in Supplementary Table 1.

### 2.4 Experimental design and acupuncture stimulation paradigm

**Figure 1:**
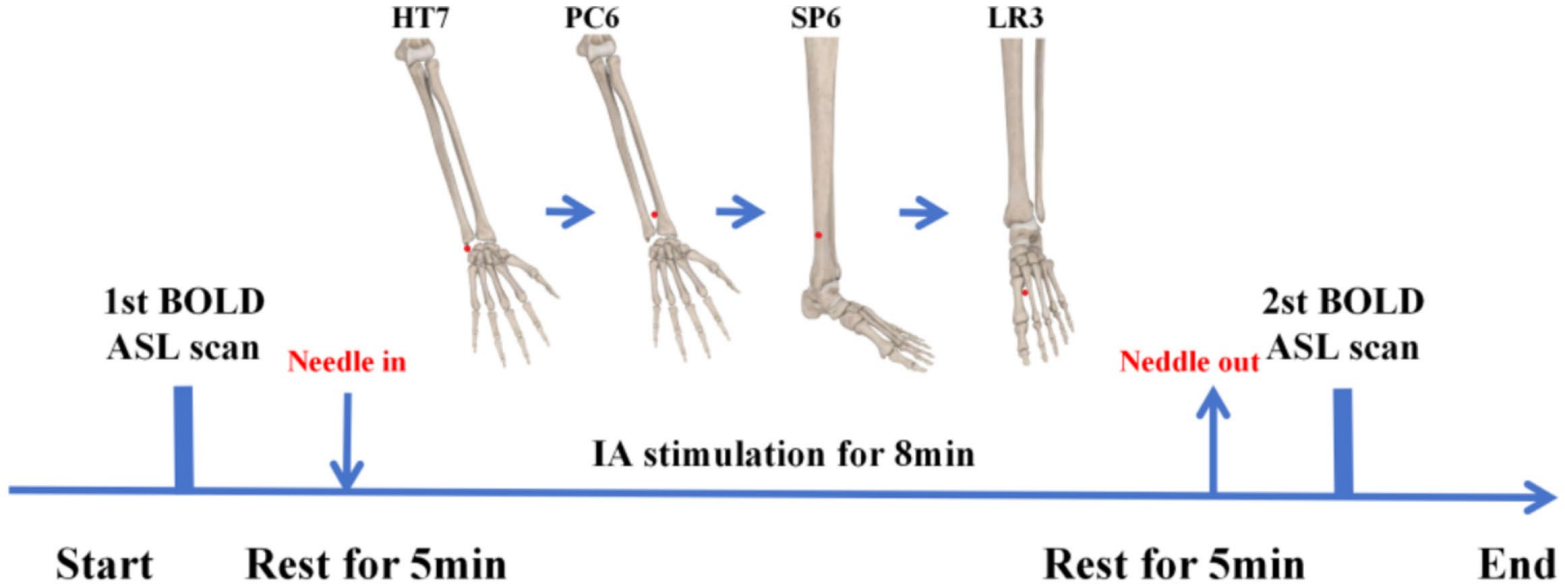
The non-repeated event-related paradigm in the MRI study.

In this MRI study, non-repeated event-related (NRER) paradigm was used to explore the effect of acupuncture (Figure 1) (Qin et al., 2008; Duan et al., 2021). HCs underwent only once scan, while patients with MDD underwent MRI scans, respectively, before and after the IA stimulation.

The selection of acupoints (PC6, HT7, SP6, and LR3) was based on our previous data mining study on acupuncture treatment for MDD, which identified these acupoints as commonly used and clinically relevant for emotional regulation (Tu et al., 2023).

Intradermal acupuncture was performed using Qingling intradermal needles 0.20 × 1.2 mm. Acupoints were stimulated bilaterally in the following order: left PC6, right PC6, left HT7, right HT7, left SP6, right SP6, left LR3, and right LR3. Manual pressing stimulation was applied for 1 min on each side at an approximate rhythm of 60 presses per minute. Thus, each acupoint received 2 min of stimulation in total, and every MDD patient underwent 8 min of continuous IA stimulation before the second MRI scan. All IA stimulation was performed by two standardized trained acupuncturists, and all MRI scans were operated by the same professional radiologist, and participants were given uniform instructions: lie flat and relaxed, keep eyes closed but stay awake.

### 2.5 Imaging acquisition and image preprocessing

Brain MRI was conducted with 3.0-Tesla General Electric scanners (Discovery MR750, GE Healthcare) equipped with an 8-channel head coil at The First Affiliated Hospital of Zhejiang Chinese Medical University. In this study, each participant underwent the acquisition of the following multi-modal MRI data: T1-weighted sagittal structural images, BOLD-fMRI images, cross-sectional T2-weighted images, ASL images, and CBF images automatically generated from the ASL images. Detailed scanning parameters and preprocessing can be found in the Supplementary material.

### 2.6 FCS analysis processing

The previously preprocessed results were used for FCS analysis by BRANT toolbox (Xu et al., 2018). The threshold of positive FC was set to 0.2 in order to eliminate negative or false correlations from background noise (Zhu et al., 2017; Yang et al., 2023). The voxel-to-voxel FC lower than 0.2 was set to zero. The FCS maps were calculated by averaging the values of functional connectivity between a given voxel and all other voxels. Then, the FCS maps were standardized into *z*-scores (zFCS) and spatially smoothed with a 6-mm FWHM Gaussian kernel.

### 2.7 CBF analysis processing

CBF maps were generated from the ASL data using the vendor’s perfusion processing pipeline based on ASL difference images and proton-density-weighted reference images with a single-compartment model. Individual CBF images in native space were spatially normalized to the Montreal Neurological Institute (MNI) space using SPM12. Specifically, for each subject the deformation field was estimated from the corresponding M0 (proton-density) image using the tissue probability map approach, and the same deformation was then applied to the subject’s CBF image to write it into MNI space. Normalized CBF maps were resampled to an isotropic voxel size of 3 mm and non-brain tissue was removed using a gray-matter mask. Subsequently, CBF maps were further standardized into *z*-scores (zCBF). Finally, zCBF maps were spatially smoothed with a 6 mm × 6 mm × 6 mm full-width at half maximum (FWHM) Gaussian kernel.

### 2.8 Voxel-wise CBF/FCS ratio analysis

To assess the cerebral blood supply relative to the strength of functional connectivity for each voxel, CBF/FCS ratio maps were computed. The ratio was derived from the original CBF (mL/100 g/min) and FCS values, followed by standardization into *z*-scores (zCBF/FCS ratio). A general linear model was constructed for zCBF/FCS ratio in SPM8, with both age and sex were included as covariates. Whole-brain voxel-wised independent *t*-test was used for intergroup comparisons and GRF correction (voxel level *p* < 0.001 and cluster level *p* < 0.05) was used for multiple comparisons correction. In addition, the differences of zCBF and zFCS between two groups at whole-brain voxel level were also explored using independent *t*-tests controlling for age and sex with GRF correction (voxel level *p* < 0.005 and cluster level *p* < 0.05).

## 3 Results

### 3.1 Demographic and clinical characteristics

Twenty MDD patients and twenty age and sex matched HC were finally included in the analysis. Demographic characteristics and the scales of depression severity are presented in Table 1. HAMD-17 scores were used to assess the severity of depression. There was no significant difference in sex, age and BMI between the MDD and HC groups (all *p* > 0.05).

**Table 1:** Demographic and clinical characteristics of the MDD patients and healthy controls.

Characteristics	MDD (*n* = 20)	HC (*n* = 20)	*p*-value
Age (years), mean (SD)	25.6 (3.7)	25.1 (5.4)	0.734^a^
Sex, No. (%)			1.000^b^
Female	7 (35.0)	7 (35.0)	
Male	13 (65.0)	13 (65.0)	
Duration of illness (months)	20.5 (9.82)	/	
BMI (kg/m^2^), mean (SD)	20.4 (1.8)	20.4 (2.7)	0.986^a^
HAMD-17 score, mean (SD)	23.2 (4.0)	1.9 (1.5)	0.000^a^

MDD, major depressive disorder; HC, healthy control; BMI, body mass index; HAMD-17, Hamilton Depression Rating Scale-17.

a Evaluated by the independent samples *t*-test.

b Evaluated by the Pearson’s chi-squared test.

### 3.2 CBF-FCS coupling changes

**Figure 2:**
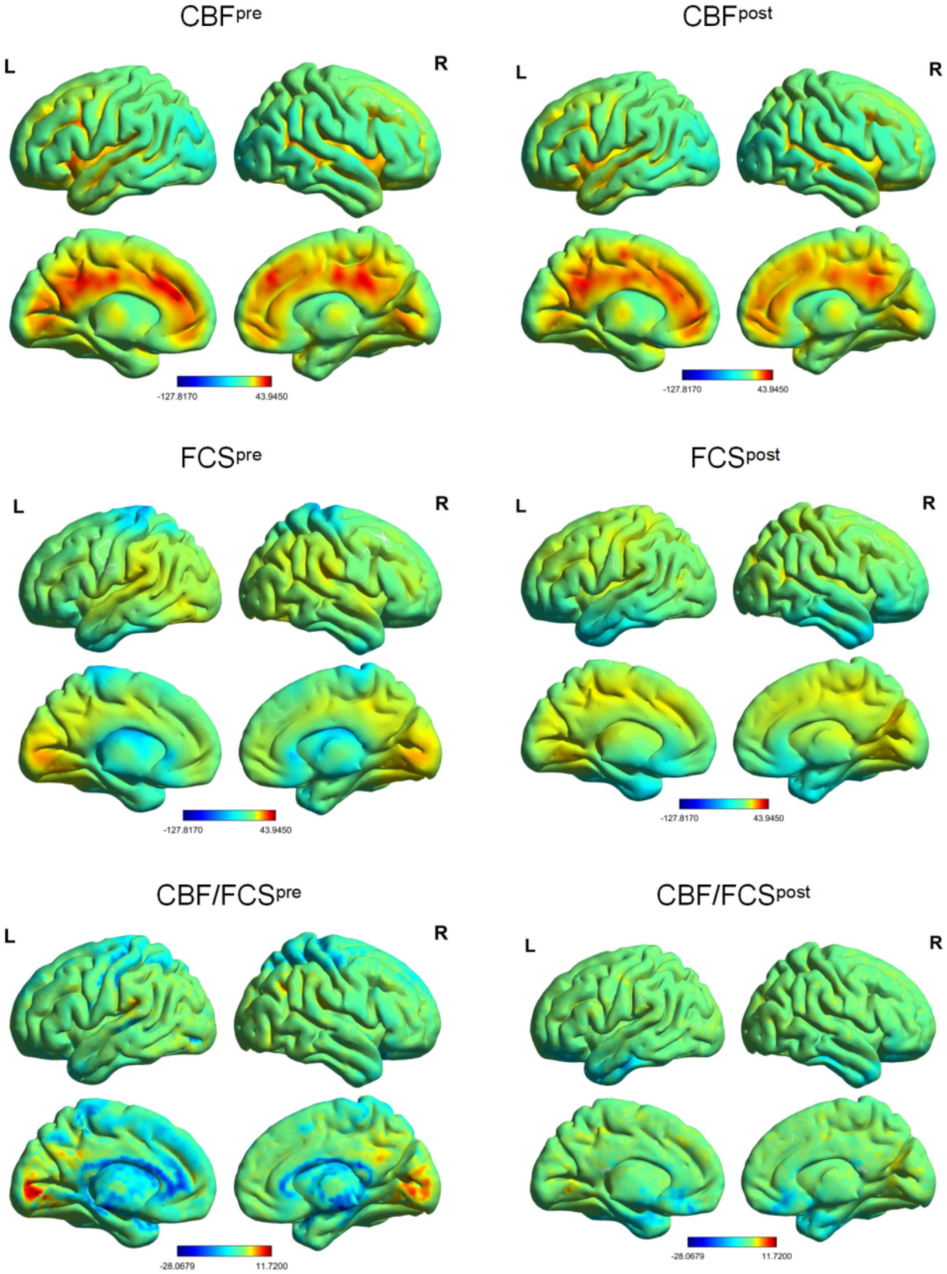
Spatial distribution maps of FCS, CBF, and CBF/FCS ratio before and after acupuncture stimulation. The FCS, CBF, and CBF/FCS ratio maps are *z*-standardized and averaged across subjects within groups. The FCS is calculated using a connectivity threshold of 0.2. CBF, cerebral blood flow; FCS, functional connectivity strength.

**Figure 3:**
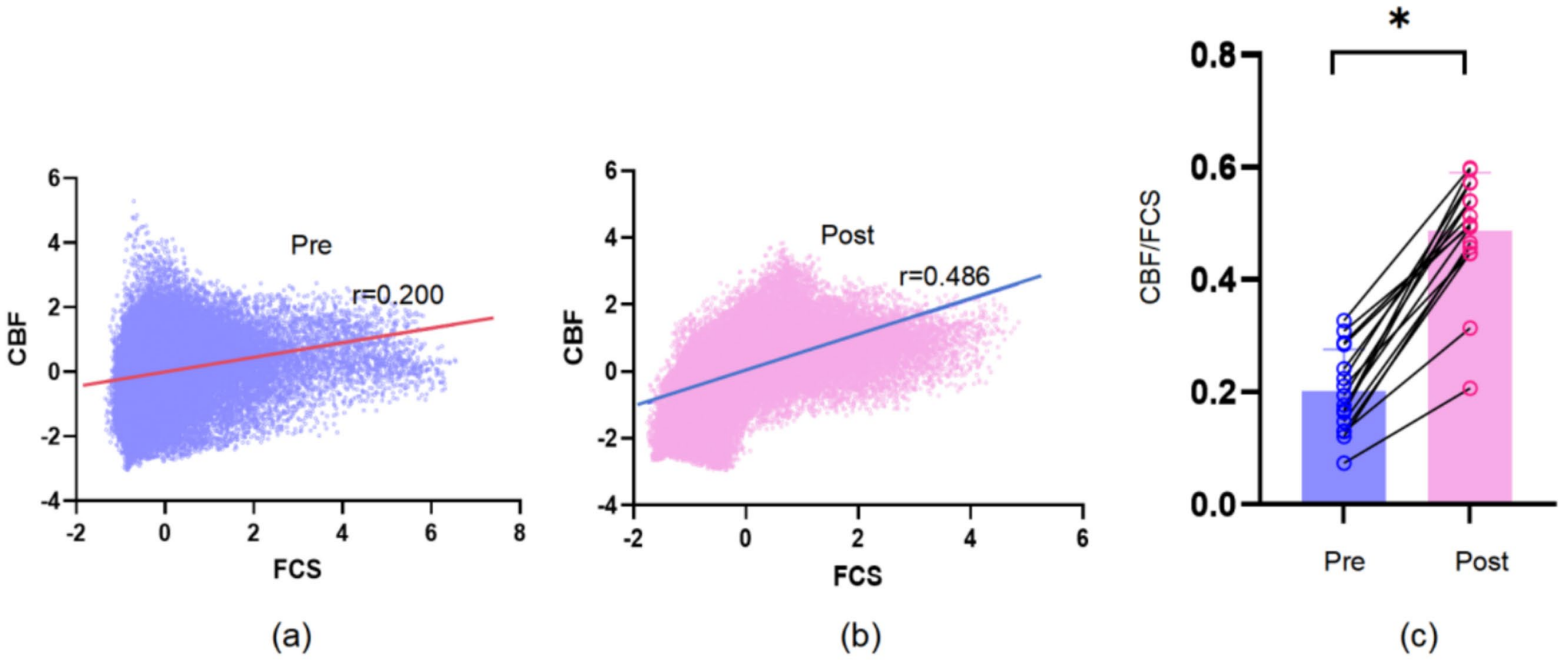
Whole gray matter level CBF-FCS coupling changes. **(a)** Scatter plots of the spatial correlations across voxels between CBF and FCS before IA. **(b)** Scatter plots of the spatial correlations across voxels between CBF and FCS after IA. **(c)** Patients showed a significantly increase in CBF-FCS coupling after IA. Significant difference was found on CBF-FCS correlation coefficients across voxels (*p* < 0.05). Error bars represent the SE. CBF, cerebral blood flow; FCS, functional connectivity strength.

The distributional patterns of CBF, FCS, and CBF/FCS ratio before and after IA are shown in Figure 2. No significant change in global CBF was observed after IA (pre: 43.6 ± 7.8 mL/100 g/min; post: 43.5 ± 8.0 mL/100 g/min; *t* = 0.52, *p* = 0.959). And global FCS showed a significant decrease (pre: 0.036 ± 0.003; post: 0.030 ± 0.010; *t* = 2.22, *p* = 0.034) in comparison to before IA. Evaluating the relationship between CBF and FCS across all voxels in the gray matter revealed a positive correlation both before and after IA (Figures 3a,b). At the subject level, CBF/FCS coupling was significantly increased following IA compared with the pre-intervention condition (pre: 0.200 ± 0.074; post: 0.486 ± 0.061; *t* = −3.51, *p* = 0.002) (Figure 3c).

Healthy controls exhibited a global CBF (controls: 43.0 ± 15.8 mL/100 g/m) and a global FCS (0.023 ± 0.003). CBF-FCS of healthy controls are 0.218 ± 0.038. Compared to MDD patient, healthy controls showed higher CBF/FCS in bilateral temporal pole, right postcentral and left precuneus, while lower CBF/FCS in left postcentral, occipital, left sup frontal, extra-nuclear (L) and right Cerebellum (Supplementary Figure 1).

### 3.3 Whole gray matter alterations of CBF/FCS ratio by acupuncture

**Figure 4:**
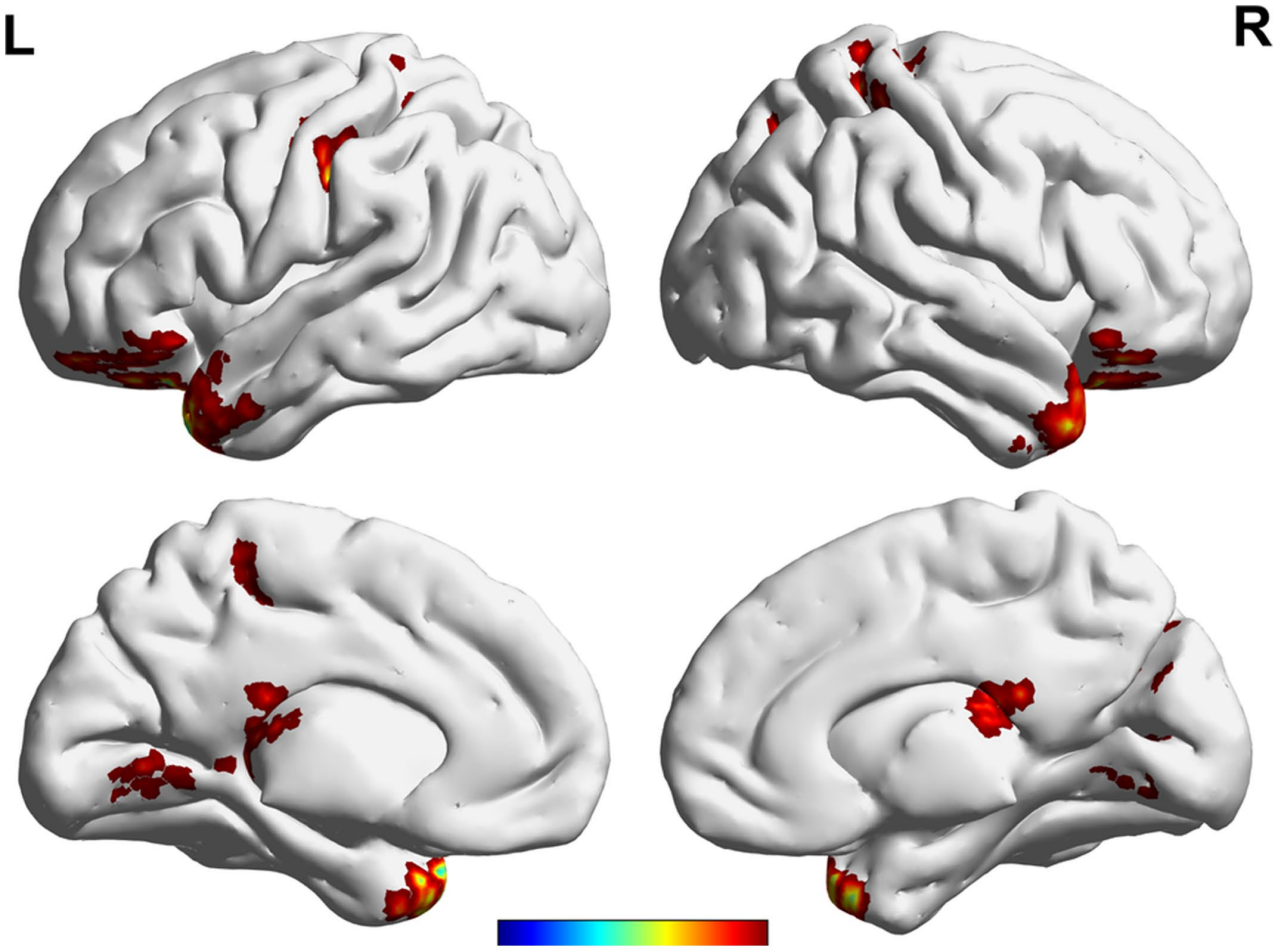
Paired differences in CBF/FCS ratio before and after IA (FDR corrected, voxel level *p* < 0.05 and cluster level *p* < 0.05). The warm and cold colors denote significantly increased and decreased CBF/FCS ratio after IA treatment. CBF, cerebral blood flow; FCS, functional connectivity strength; L, left; R, right.

Voxel-wise whole gray matter analysis revealed significant alterations in the CBF/FCS ratio after acupuncture treatment (Figure 4). Significant clusters were observed in the bilateral superior temporal pole, right cerebellar anterior lobe, right occipito-temporal cortex, left superior occipital cortex, bilateral postcentral gyrus, bilateral precuneus, left superior frontal gyrus. Peak coordinates, voxel sizes, and peak intensities for all significant clusters are summarized in Table 2.

**Table 2:** Alterations of CBF/FCS coupling after intradermal acupuncture.

Cluster	Voxels	Peak MNI (*x*,*y*,*z*)	Peak intensity	Peak label
1	204	−30, 21, −30	15.257	Temporal_Pole_Sup_L
2	119	30, 24, −27	13.422	Temporal_Pole_Sup_R
3	55	9, −54, −3	15.705	Cerebelum_4_5_R
4	63	−24, −36, 9	7.089	Extra-nuclear (L)
5	80	30, −66, 21	10.071	Occipito-temporal (R)
6	42	−18, −69, 33	7.058	Occipital_Sup_L
7	32	−45, −18, 51	8.854	Postcentral_L
8	51	24, 63, 51	7.492	Precuneus_R
9	44	−15, 6, 54	7.270	Frontal_Sup_L
10	118	−15, −42, 66	8.561	Precuneus_L
11	34	30, −36, 63	8.383	Postcentral_R

## 4 Discussion

To our knowledge, this is the first study to investigate changes in CBF/FCS coupling associated with immediate central effects of acupuncture in patients with MDD. In this study, we examined how intradermal acupuncture modulates cerebral CBF/FCS coupling in patients with MDD. By integrating resting-state cerebral blood flow and functional connectivity strength, we identified widespread alterations in the CBF/FCS ratio following acupuncture, involving sensorimotor, parietal, frontal, and cerebellar regions. These findings suggest that acupuncture modulates large-scale brain systems related to sensory processing, motor control, and cognitive functions.

The coupling between CBF and FCS is thought to reflect the relationship between regional perfusion and large scale neural integration, representing an important aspect of neurovascular coupling (Liang et al., 2013). This coupling depends on the integrity of neurovascular unit (neurons, glial cells, and vascular components) (Zhu et al., 2017; Howarth, 2014). In the present study, the CBF/FCS ratio was used as an index of the relative relationship between cerebral perfusion and functional connectivity strength. It may reflect a shift in the coordination between metabolic supply and functional network integration, arising from changes in either component or both. In this study, we observed an immediate increase in CBF/FCS following acupuncture, indicating that acupuncture may enhance the coordination between local metabolic support and functional network integration. The observed alterations in CBF/FCS coupling induced by IA may promote this balance by enhancing the synchronization between local brain activity and metabolic demands, thereby facilitating more efficient neural processing. This neurovascular coupling may contribute to the normalization of brain function in regions that are critical to mood regulation, sensory processing, and cognitive control.

Significant alterations in CBF/FCS were observed in precuneus and postcentral gyrus, indicating the potential role of these regions in processing sensory-motor integration and emotional regulation which are key aspects of depression (Yan et al., 2019). The precuneus is a core hub of the default mode network and is closely associated with self-referential processing and rumination, which are central features of MDD (Zhou et al., 2020). Therefore, altered coupling in this region may reflect modulation of maladaptive self-focused cognitive activity. The modulation of perfusion-connectivity coupling in these areas may reflect a reorganization of sensorimotor processing, potentially restoring a more efficient balance between local neural activity and metabolic support. Such a mechanism is consistent with clinical observations that acupuncture can alleviate somatic symptoms and improve bodily awareness in depressed patients. Similarly, the postcentral gyrus is increasingly recognized as being involved not only in somatosensory processing but also in the integration of bodily perception and emotional experience. Altered coupling in this region may therefore be related to the abnormal bodily and affective experiences often observed in patients with depression.

Alterations were also detected in frontal regions, including the superior and medial frontal gyrus. These areas play key roles in cognitive control, emotional regulation, and decision-making, functions that are often compromised in depression (Pan et al., 2019). Changes in perfusion-connectivity coupling in frontal cortex may therefore reflect enhanced regulatory capacity following acupuncture. Rather than simply increasing or decreasing activity, acupuncture may facilitate a more coordinated relationship between functional integration and cerebral blood supply, supporting more stable frontal network function (Stanca et al., 2023).

In addition, the cerebellar involvement was observed. Although traditionally associated with motor coordination, the cerebellum is increasingly recognized for its role in affective and cognitive processing. Previous studies have reported cerebellar abnormalities in MDD (Stanca et al., 2023). Our findings further support the idea that cerebellar circuits participate in the therapeutic effects of acupuncture. The modulation of CBF/FCS coupling in these regions may indicate adjustments in cortico-cerebellar communication during treatment. Such changes may contribute to improved emotional stability and cognitive coordination in patients with depression.

Compared with conventional acupuncture modalities, such as manual acupuncture and electroacupuncture, IA is characterized by sustained, low-intensity stimulation that can be maintained over an extended period. This continuous stimulation pattern differs from the more transient and relatively higher-intensity stimulation which applied in conventional acupuncture and may induce more gradual and stable modulation of brain function. In addition, IA may offer practical clinical advantages, particularly better patient compliance, because it is minimally invasive and can provide continuous stimulation with less interference in daily activities. Previous work from our group has also provided evidence supporting the clinical efficacy of IA in patients with MDD, which further supports its therapeutic relevance (Wu et al., 2025).

Several limitations of the present study should be acknowledged. First, the sample size was relatively modest, which may limit the statistical power and generalizability of the findings. Future studies with larger cohorts are needed to validate the robustness and reproducibility of the observed CBF/FCS coupling alterations. Second, the current study focused on the immediate neural effects of intradermal acupuncture, as imaging data were acquired shortly after a single acupuncture session. While accumulating evidence suggests that the long-term therapeutic benefits of acupuncture may arise from the accumulation of repeated immediate effects (Wang X. et al., 2025), the present design does not allow direct inference regarding sustained or longitudinal neural changes. Future longitudinal studies incorporating multiple treatment sessions and follow-up assessments are therefore warranted to elucidate the long term neurobiological mechanisms underlying acupuncture treatment in MDD.

Third, an important limitation is the absence of sham acupuncture control group. As a non-pharmacological intervention, acupuncture is susceptible to placebo effects and non-specific influences related to expectancy and somatosensory stimulation. Notably, even acute interventions can induce rapid placebo-related neural responses through expectancy-driven and top-down regulatory mechanisms, which may modulate cerebral blood flow and functional connectivity within a short time window. Therefore, the observed changes in CBF/FCS coupling cannot be unequivocally attributed to the specific effects of intradermal acupuncture, but may instead reflect a combination of specific and non-specific neural modulation. Future studies incorporating sham acupuncture or other control conditions are warranted to disentangle these effects and further clarify the underlying mechanisms.

## 5 Conclusion

This study provides neuroimaging insights into the underlying mechanisms of intradermal acupuncture in MDD. By examining changes in both cerebral blood flow and functional connectivity, we found that acupuncture modulates the relationship between brain perfusion and neural activity across several brain regions involved in sensory processing, emotional regulation, and cognitive control. These results suggest that acupuncture may improve brain network function by enhancing neurovascular coupling, contributing to its therapeutic effects in MDD. Future studies with larger samples and longitudinal designs are needed to explore the long-term neurobiological mechanisms of acupuncture in depression.

## Data Availability

The raw data supporting the conclusions of this article will be made available by the authors, without undue reservation.
