# Supplementary material for: Cerebral blood flow and functional connectivity immediate changes following intradermal acupuncture in major depressive disorder
Source: Front Neurosci. 2026 Apr 17;20:1805907. doi: 10.3389/fnins.2026.1805907 (PMC13132869; doi:10.3389/fnins.2026.1805907)
Supplement: Supplementary file 1 [file Data_Sheet_1.DOCX]

Supplementary Material

# 1. Detailed scanning parameters

Magnetization-prepared rapid gradient echo sequence was used to acquire high resolution 3D T1-weighted sagittal images. The parameters were as follows: repeat time (TR) = 8.2 ms; echo time (TE) = 3.2 ms; flip angle (FA) = 12 degree; matrix size = 256 mm*256 mm; slice thickness = 1 mm; 152 slices. Resting-state BOLD fMRI data were collected using a gradient-echo single-shot echo planar imaging sequence with the following parameters: TR = 2000 ms; TE = 35 ms; FA = 90 degree; matrix size = 64 mm*64 mm; slice thickness = 5 mm, 6960 slices; Cross-sectional T2-weighted images were acquired to exclude organic brain lesions with the following parameters: TR = 5813.5 ms; TE = 84.1 ms; slice thickness = 4 mm, 20 slices; The parameters of ASL were as follows: TR = 5029 ms, FA = 90 degree, matrix size =128 mm*128 mm, slice thickness = 4 mm, 72 slices, and cerebral blood flow (CBF) generated automatically from ASL images.

Two experienced radiologists assessed image quality to ensure that there were no obvious head movement artifacts or abnormal signals, and then classified and processed the scanned data. Magnetic resonance data were preprocessed using the RESTPLUS toolbox^27^ and SPM8.0 (http://www.fil.ion.ucl.ac.uk/spm) in MATLAB 2020a, mainly includes the following steps: 1) convert DICOM format data to NII or NIFTI formats; 2) remove the first 10 time points to reduce the initial instability of the magnetic field and the impact of the subjects’ discomfort with image acquisition; 3) slice timing, which corrects for data bias introduced by MR scans; 4) realign, head movement files were checked, and subjects with head movement displacement ≤ 3 mm or head movement Angle ≤ 3° were retained; 5) Spatial normalization was performed by segmenting the images and normalizing them to the standard MNI space; 6) regression covariates to eliminate the influence of head movement, cerebrospinal fluid, and white matter signals; 7) a temporal band-pass filter between 0.01- 0.10 Hz after regression was used to minimize the influence of heartbeat, breathing signals and the noise of the scanner itself; 8) smooth.

| **sTable 1**. Inclusion and Exclusion Criteria | |
| --- | --- |
| **Inclusion Criteria** | **Exclusion Criteria** |
| - Patients diagnosed with MDD according to the International Classification of Disease-10 (ICD-10); HAMD-17≥17; HCs with HAMD-17<7 and no history of any psychotic disorders; - Aged between 18 and 60 years (no limitation on gender); - Administration of SSRIs at least 6 weeks; - Participates undergoing MRI should be right-handed and free of traumatic brain injury, claustrophobia or metal implants; - Written informed consents are obtained by participates. | - **ICD-10 diagnoses: schizophrenia, bipolar disorder, manic episode or other psychotic disorders; alcohol and drug addiction; a current substance use disorder and lifetime history of substance abuse;** - **Significant skin lesions, severe allergic diseases, tumors, and severe or unstable internal diseases involving the cardiovascular, digestive, endocrine, or hematological system;** - **Positive suicidal tendency;** - **Allergy to adhesive tape, fear of intradermal acupuncture;** - **Pregnancy and lactation;** - **Mental retardation and difficulty cooperating with doctors;** - **Previously treated with intradermal acupuncture or participating in other clinical trials.** |
| MDD, major depressive disorder; HCs, health controls; SSRIs, selective serotonin reuptake inhibitors; | |

| **sTable 2. Acupoints Locations** | | |
| --- | --- | --- |
| **Acupoints** | **Code** | **Localization** |
| Shenmen | HT7 | Located on the palmar ulnar end of the transverse crease of the wrist and on the radial aspect of the tendon of the ulnar flexor. |
| Neiguan | PC6 | Located 2 cun above the transverse wrist crease between the tendons of the palmaris longus and flexor carpi radialis. |
| Sanyinjiao | SP6 | Located 3 cun directly above the tip of the medial malleolus, on the posterior border of the tibia. |
| Taichong | LR3 | Located on the dorsal side of the foot and sunken behind the first metatarsal space. |


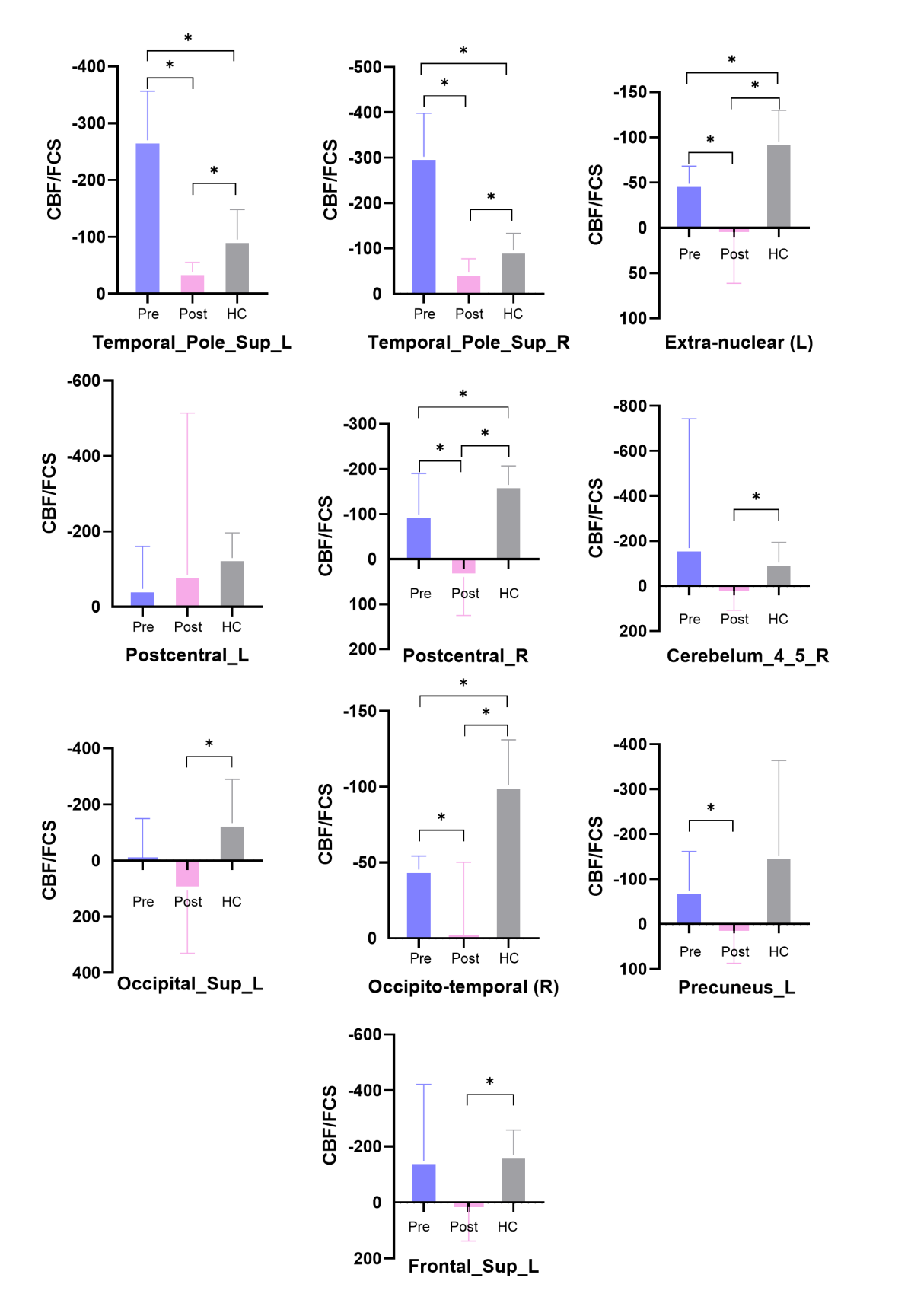


# sFigure1. ROI-based CBF/FCS comparisons between Pre/Post-IA MDD patients and healthy controls.
